# Health-Related Quality of Life of Patients with Marfan Syndrome—Polish Study

**DOI:** 10.3390/ijerph19116827

**Published:** 2022-06-02

**Authors:** Agnieszka Trawicka, Aleksandra Lewandowska-Walter, Mikołaj Majkowicz, Robert Sabiniewicz, Lidia Woźniak-Mielczarek

**Affiliations:** 1 Department of Pediatric Cardiology and Congenital Heart Diseases, Medical University of Gdansk, 80-211 Gdansk, Poland; sabini@gumed.edu.pl (R.S.); lidiawozniak@yahoo.com (L.W.-M.); 2 Department of Psychology, Gdansk University of Physical Education and Sport, 80-336 Gdansk, Poland; 3 Institute of Psychology, University of Gdansk, 80-339 Gdansk, Poland; aleksandra.lewandowska-walter@ug.edu.pl; 4 Institute of Health Science, Pomoranian Academy in Słupsk, 76-200 Słupsk, Poland; mmajk@gumed.edu.pl

**Keywords:** Marfan syndrome, quality of life, rare genetic diseases, health-related quality of life, aortic aneurysm

## Abstract

Background: Despite extensive knowledge about the quality of life of people suffering from rare diseases, data on patients with Marfan syndrome (MFS) are scarce and inconsistent. Hence, the problem of assessing the quality of life (QOL) and its relationship with the assessment of which ailments are the most burdensome for these patients is still open. Aim: Comparison of the quality of life of patients with MFS and determination as to which of the reported complaints in patients with MFS are related to the QOL of patients. Methods: The study included 35 patients with MFS and 35 healthy controls, matched for gender and age. In the study, the questionnaire of quality of life assessment SF-36 was used to assess the level of health-related quality of life, as well as an interview of the most severe symptoms reported by patients with MFS. Results: The level of the physical dimension of the QOL (*p* < 0.001) and limiting of roles due to physical health (*p* = 0.002), as well as the level of general index of the QOL (*p* < 0.001), were statistically significantly lower in MFS patients when compared to controls. People from both studied groups do not vary in the scope of pain, vitality, social functioning, limiting the roles due to emotional problems, and state of mind but also in the mental dimension of the health-related quality of life (HRQL). Additionally, there has been a correlation between HRQL and the subjective assessment of the effects of orthopedic, ophthalmic, and cardiological problems in life, as well as lower exercise tolerance in the evaluation of people with MFS and QOL in most areas. Conclusions: Patients with MFS present a reduced QOL in the areas of physical functioning, limiting roles due to physical health, general feeling of general health, the physical dimension of the HRQL, and the general index of the QOL; in these areas, they require careful evaluation, as well as medical and psychosocial assistance.

## 1. Introduction

Marfan syndrome is a genetically conditioned disease of the connective tissue. It affects multiple organs and systems. The most common triad of symptoms concerns the skeletal system, cardiovascular system, and sight. It rarely concerns the respiratory and nervous system, skin, muscles, and fat tissues. However, the abnormalities concerning the cardiovascular system present a risk of shortening life [1,2]. Marfan syndrome is characterized by a great changeability in a phenotypical image, but also in a variability of the symptom intensity when it occurs in relatives [3].

Marfan syndrome fulfills the criteria of a rare disease (rare diseases are those which occur with a frequency below 1 per 2000 births) [4]. It is estimated that Marfan syndrome occurs in 4–10 people per 100,000; however, some sources have found up to 17–20 cases per 100,000 [5,6,7]. There is a relatively small number of studies concerning the quality of life and its conditioning in people with rare genetic diseases, especially concerning people with Marfan syndrome. Available foreign reports concerning the quality of life of people with Marfan syndrome are not always coherent, and the issue of conditioning of the quality of life is hardly raised and requires further analysis.

A review of available scientific articles in English concerning the quality of life in adults with Marfan syndrome [8,9,10,11,12,13,14,15,16,17,18,19,20,21,22] has allowed us to select a group of scientific articles on the topic of the quality of life conditioned by a health status in Marfan syndrome. As a result of the analysis and interpretation of the results of the studies, we selected the largest group of studies conducted by means of the SF-36 questionnaire to examine the quality of life conditioned by a health status [8,10,11,12,13,17,19].

During the next stage, scientific works concerning the most numerous groups of patients with clinically diagnosed Marfan syndrome were selected [11,13,17]. The results of these studies revealed that the quality of life conditioned by a health status in Marfan syndrome was lowered. The analysis of the results in this group revealed the differences in the scope of the areas of quality of life in which considerable reduction of the correlates of the quality of life was noticed.

Studies [11,13,17] revealed that people with Marfan syndrome in comparison with the general population have a lower quality of life in the area of mental health. People with Marfan syndrome assess their physical functioning higher than their mental functioning. A group of respondents with Marfan syndrome on the basis of the SF-36 scale assess themselves better on the scale of physical functioning and limiting a role due to physical reasons, as well as social functioning; however, the lowest assessment concerns the scale pain, limiting a role—emotional problems, vitality, and general health. This means that there is a significant connection between a clinical image of Marfan syndrome and a psychosocial adjustment and mental aspects of quality of life. Furthermore, elder age and male gender were significantly connected with a lowered quality of life in the area of mental functioning.

Another study by Rand-Hendriksen, Johansen [17] shows that quality of life connected with health in people with Marfan syndrome is lowered in a comparable degree to the quality of life of people with other chronic diseases, and is not connected with biomedical factors. People with Marfan syndrome in comparison with a control group have reached lower results on all SF-36 sub-scales in the area of physical health and on the sub-scales of vitality and social functioning; therefore, it affected the lower result on the scale of general health. In comparison with people with chronic uveitis, cystic fibrosis, hypertrophic cardiomyopathy (the risk of sudden death and cardiovascular problems), people with Marfan syndrome revealed a lower quality of life connected with health. Not including a group with Behcet’s disease, people with Marfan syndrome have much lower results than comparable groups in the scope of social functioning and the lowest results in all groups in the scale of vitality. Low vitality in people with Marfan syndrome may be connected with complaints connected with tiredness and increased fatigue during physical activities. On the sub-scales connected with physical functioning in SF-36, people with Marfan syndrome reported a greater intensity of physical pain in relation to comparable groups, with the exception of Behcet’s disease. What was surprising for the authors of the article was the fact that people with Marfan syndrome have lower results in limiting a role—physical problems than people with cystic fibrosis. It is assessed that cystic fibrosis is a more severe disease than Marfan syndrome, taking into consideration worse functioning and shorter life expectancy. It is possible that required daily care of these patients may be supportive for them, while people with Marfan syndrome are left alone in their daily life [17].

In the scope of the correlates of quality of life, no significant relationship between age, sex, BMI, and the SF-36 sub-scales was noticed. However, the older the patient gets, the level of pain increases and the results in the scale of physical functioning in the SF-36 decrease. Having a member family member with Marfan syndrome in the first line was connected with higher results in the scale of limiting a role due to emotional problems. Fulfilling Ghent criteria in the scope of short-sightedness was related to the above mentioned results in the scale of general health and physical functioning. There has been no relationship between the results on the SF-36 scales and such medical variables as extension of the aorta, having undergone operations, beta blockers, reduced vision acuity, joint hypermobility, fulfillment of five major criteria in Ghent scale, and the number of fulfilled Ghent criteria [17].

The study by Goldfinger [12] showed that the quality of life of people with Marfan syndrome connected with health is lowered in comparison with the general population. Unlike the results of the study by Fusar-Poli [11], where the indicator—mental health in SF-36 was lowered, in this study, the indicator—physical health in SF-36, the studied group with Marfan syndrome achieved the lowest results. Goldfinger’s [12] study was conducted on a much larger group, consisting of 389 people, while Fusar-Poli [11] studied 36 people. The predictors of a better quality of life conditioned by a health status distinguished by Goldfinger et al. [12] were: higher education, being in a relationship, higher income, private health insurance, having a full-time job, moderate alcohol intake, lower number of operations, lower number of concomitant diseases, lack of depression, and less serious symptoms of Marfan syndrome. In a multi-dimensional analysis of the variance, a status of insurance and employment was a statistically significant predictor of a quality of life. What is significant in this study is the conclusion that the quality of life connected with health was not significantly connected with the factors connected with health condition and symptoms and their intensity in Marfan syndrome. The quality of life was conditioned by socioeconomic factors.

Moon et al. [13] undertook the analysis of the quality of life of patients with Marfan syndrome with the use of structural equation modeling. As a result of the analyses, they presented that a lowered quality of life is connected with: older age, lower level of education, lower economic status, lower social support, greater number of cardiological surgeries, higher level of anxiety, higher level of depression, higher level of tiredness, higher level of pain, and unfavorable body image; however, the quality of life was less connected with demographic factors. In the context of the presented analyses, one may state that in a structural modeling, social support, factors connected with the disease and bio-behavioral factors in 72.4% explained/determined the quality of life of people with Marfan syndrome. Behavioral factors in 39.2% explained/affected the level of social support, and demographic factors explained only 12.4%.

The analysis of the available literature and the research results discussed above allowed for the formulation of the research aim. Our purpose was to define the specificity of functioning of people with Marfan syndrome in comparison with a group of healthy people and the assessment of the relationship between the quality of life of people with Marfan syndrome with the indicators of health status. It was assumed that 1) there are differences between a group of patients with Marfan syndrome and a group of healthy people in the scope of the assessment of the quality of life, and 2) there is a relationship between the assessment of the quality of life and the health status in people with Marfan syndrome.

## 2. Materials and Methods

### 2.1. Participants

The study included people admitted to the Clinic of Pediatric Cardiology and Congenital Heart Defects at the University Clinical Center in Gdańsk, who gave their consent to participate in the study. The basic criteria of inclusion were: being above the age of 18, diagnosis of Marfan syndrome, and medical assessment made by a doctor (not confirmed by genetic data). The exclusion criteria included: concomitant somatic diseases and consciousness disorders, and disorders of cognitive function that would make it impossible to complete the questionnaires. A group of healthy people were selected by pairs with regards to sex and age. Inclusion criteria included lack of diagnosis of chronic disease and not being under constant medical care of a specialist; exclusion criteria were similar to those in the group of people with Marfan syndrome: consciousness disorders and disorders of cognitive functions. Demographic data on the studied groups are presented in Table 1. and clinical data concerning people with Marfan syndrome are presented in Table 2.

With the adopted significance level of *p* < 0.05, a statistically significant difference was only found in the financial situation (χ^2^ = 11,114; df = 2; *p* = 0.003).

### 2.2. Assessment

In the study, the questionnaire of quality of life assessment SF-36 was used [23]. It is one of the most commonly applied questionnaires by John Ware used to assess the quality of life in people with somatic diseases. The British Medical Journal recognized the SF-36 questionnaire as the most well-checked and widest used scale to assess the quality of life connected with health [23]. The SF-36 scale achieved a high level of reliability—Cronbach’s alpha—for particular parameters of health, which were over j 0.80 [24]. The questionnaire consists of 36 positions and allows the diagnosis of 8 aspects of health: 1. Physical functioning, 2. Limiting the roles due to physical health problems, 3. Pain, 4. Social functioning, 5. Self-feeling (psychological stress and psychological well-being), 6. Limiting the roles due to emotional problems, 7. Vitality (energy/tiredness), and 8. General concept concerning the issue of health—general health. Every scale consists of 2–10 test positions. Particular sub-scales are included in the content of general scales: physical dimension of the quality of life, mental dimension of the quality of life, and index of the quality of life.

In order to assess the health status, Ghent criteria were used. These criteria assess the incidence of ascending aorta dilatation occurring and ectopia lentis based on the family history. In case of any diagnostic doubts, other features of Marfan syndrome are taken into consideration and included in the systemic scale. A patient fulfills diagnostic criteria when he or she gets at least 7 out of 20 possible points [25]. A systemic scale includes the following features of Marfan syndrome: thumb symptom, wrist symptom, pigeon chest, pectus excavatum, chest asymmetry, flat foot, metatarsal deformation, protrusion of head of femur, pneumothorax, reduced relation of the upper body part to the lower part, increased relation of the arms span, scoliosis, kyphosis, limited strengthening of an elbow (<170°), dysmorphic facial features, stretch marks, myopia, and mitral valve prolapse [25].

The average number of points obtained by the group studied was 7.63, while the maximum number of points was 14. Reduced relation of the upper body part to the lower part and increased relation of the arms span to the height with the absence of scoliosis was noticed almost in the whole group. Other symptoms that often occurred were scoliosis and kyphosis (26 people), and the asymmetry of the chest and thumb symptom were observed in 22 people. Mitral valve prolapse concerned 20 people, and a wrist symptom was noted in patients. Pectus excavatum, flat foot, dysmorphic facial features, stretch marks, and myopia occurred in approximately half of the group, and metatarsal deformation occurred in 7 people.

The rarest symptom included in the Ghent scale concerned the limited strengthening of an elbow (<170°) (3 people) and pneumothorax in 1 person. Medical data above mentioned concerning Ghent criteria in people with Marfan Syndrom are presented in Table 3.

### 2.3. Organization and the Course of the Study

Meetings with the patients with Marfan syndrome took place in the Clinic of Pediatric Cardiology and Congenital Heart Defects at the University Clinical Center in Gdańsk and happened gradually. The project received a positive opinion from the Independent Bioethics Commission for Research. People with Marfan syndrome were examined by doctors of different specialties (depending on the specificity of the symptoms). Within the framework of the participation in the study, there was a possibility to have a psychological consultation for the patient and/or his/her family. During the talk with a psychologist, the patients were asked for a consent to participate in the psychological part of the project. Participation in the study was voluntary, and the studied person could refuse participation in the study, which was confidential. Patients were acquainted with the general aim of the study, assured about the anonymity of the study, and informed about the possibility of getting feedback. A psychologist accompanied the people who were filling in the questionnaires, answered all possible questions, and also offered the possibility of a conversation after completing the research part. Filling in the whole set of questionnaires takes between 30 to 60 min. Due to the nature of the medical study, the patients have a few appointments in the clinic, which allows for the possibility of conducting a paper and pencil psychological examination in two or more stages. Thanks to this, the emotional and cognitive burden of the patients was minimized.

## 3. Results

The results of the analysis between studied groups in the scope of the quality of life conditioned by the health status assessed by SF-36 are presented in Table 4.

The results presented in Table 5 concern the differences on the level of the quality of life between the groups. The differences were observed in the scope of physical functioning (*p* < 0.001) and limiting roles due to physical health (*p* = 0.002), and general feeling of general health (*p* < 0.001)—people with Marfan syndrome evaluate their quality of life lower in the given areas. The differences also appeared on the level of physical dimension of the quality of life (*p* < 0.001) and on the level of the general index of the quality of life (*p* < 0.001). On the basis of the obtained results, it can be stated that people with Marfan syndrome present a lower quality of life than people in the comparative group. On their basis, it can be stated that people from both studied groups do not vary in the scope of: pain, vitality, social functioning, limiting the roles due to emotional problems, and state of mind, as well as the mental dimension of the quality of life.

Based on the obtained results included in the table, significant differences between the level of the quality of life with regards to gender were noted only in the group of people with Marfan syndrome; these differences were not noted in the group of healthy people. In the scope of physical functioning, general health, vitality, and state of mind and also in physical and mental areas of the quality of life, men with Marfan syndrome assessed their quality of life higher than women with Marfan syndrome.

In order to obtain a subjective evaluation of the effect of orthopedic, ophthalmic, cardiological problems, and lower exercise tolerance on the quality of life of people with Marfan syndrome, an analysis of the correlation of these variables with the assessment of the quality of life was made; the data are presented in the table below.

Based on the analysis of the data included in Table 6, there was a correlation between a lower quality of life in the areas of physical functioning, general health, vitality, social functioning, and state of mind and also physical and mental dimensions of the quality of life, and in the general index, and a higher evaluation of a disorganizing effect of the orthopedic problems on the life of an individual with Marfan syndrome. There is a correlation between a lower quality of life in the area of vitality and a higher evaluation of a disorganizing effect of the ophthalmic problems on the life of an individual with Marfan syndrome. A higher evaluation of a disorganizing effect of the cardiological problems on the life of an individual with Marfan syndrome co-occurs with a lower evaluation of the quality of life in the area of limiting roles due to physical problems. A higher evaluation of a disorganizing effect of a lower exercise tolerance on life co-occurs with a lower evaluation of the quality of life in the areas of physical functioning, limiting roles due to physical problems, and social functioning but also in the physical dimension of the quality of life, as well as the general indicator of the quality of life.

Based on the data included in the Table 7, no correlation between the obtained number of points in a systemic Ghent scale and a quality of life of people with Marfan syndrome was noted.

## 4. Discussion

The first aim of the study was to define the quality of life of people with Marfan syndrome in comparison with the group of healthy people (not suffering from any chronic diseases). The hypothesis which we put forward in this area was verified positively. In the area of the quality of life conditioned by health status, it was observed that people with Marfan syndrome evaluate their quality of life lower in the scope of physical functioning, limiting roles due to physical problems, general health but also physical dimension of health, as well as the general index of the quality of life in comparison with the group of healthy people.

This means that the differences in the scope of the quality of life between the studied groups are visible, especially in the areas concerning physical functioning. People with Marfan syndrome in comparison with the group of healthy people notice that they have more limitations in activity, they often have negative convictions about their health, they more often predict that their health will deteriorate, they have a stronger intensity of pain, they are tired more often, and they generally think that their evaluation of health will turn out to be negative. While comparing the obtained results to other reports, it can be stated that they are in accordance with those presented by Rand-Hendriksen et al. [17], in which people with Marfan syndrome have lowered results on all the sub-scales concerning physical health, but also by Goldfinger et al. [11], as well as by Benninghoven et al. [8], where physical functioning was lowered in comparison with the general population. A greater risk of impaired quality of life in patients with Marfan syndrome has also been confirmed by cross-sectional studies by Andonian et al. [26].

In order to assess the relationship between gender and the assessment of the quality of life of people with Marfan syndrome, an analysis concerning the scope of the quality of life between men and women was conducted. It was noted that in the scope of physical functioning, general health, vitality, and state of mind, as well as physical and mental areas of the quality of life, men with Marfan syndrome evaluate their quality of life higher than women with Marfan syndrome, which is consistent with results from Thijssen et al. [11]. Nevertheless, the obtained result was different in relation to other results—in the study by Foran et al. [10], there were no statistically significant differences connected with gender, and in the study by Fusar-Poli et al. [11], men had a significantly lower quality of life in the area of mental functioning. It is possible that men who participated in our study, when faced with the disease, activated the mechanism of self-enhancement—as a result of a disease, a sick patient gains a higher self-esteem. At present, however, it is thought that this phenomenon has an illusory character, does not prove the authentic change, and may present a denial style of coping [27,28]. This fact is difficult to interpret, as it is not possible to assess whether it is a matter of a real health status or only its perception. Further research in this area is needed.

While searching for the answer to the research question concerning the differences in the scope of the quality of life in the group of patients with Marfan syndrome with regards to the clinical image, a comparison of means in particular areas of the quality of life taking into consideration the presence or absence of a given clinical symptom was made. During the analysis of the data from the results of our study concerning the correlation of various areas of the quality of life and a subjective patient’s evaluation of the effect of particular health problems on his/her life, it was noted that the subjective evaluation of the effect of the health status presents a great number of dependencies with various areas of the quality of life. Therefore, the hypothesis was confirmed.

The assessment of a greater disorganizing effect of the orthopedic problems on life is related to lowering the quality of life in its many areas (physical functioning, general health, vitality, state of mind), but also to each dimension: mental, physical, as well as lowered index of the quality of life. The studies show that people who do not have the limitations of the mobility of joint present a higher quality of life in the area of limiting roles—physical problems. It seems that the orthopedic symptoms and their disorganizing effect on life is connected with lowering quality of life in so many areas that, for this reason, they may constitute a source of pain in people with Marfan syndrome. Pain has a significant negative influence on the quality of life, as it is often connected with insomnia and depression [29]. Taking into consideration the fear-avoidance model, one may put forward a hypothesis that people with Marfan syndrome who experience pain connected with orthopedic problems avoid doing daily activities, which is reflected in the lowered quality of life [30].

In the study by Peters et al. [16], 90% of those studied reported the experience of pain every day; in the study by Speed et al. [20], 89% of those with Marfan syndrome experienced pain and had lower results on the scale of mental functioning. Schoormans et al. [19] obtained the results confirming the significant correlation of the occurrence of scoliosis with a lower quality of life in the area of physical health.

A higher evaluation of a disorganizing effect of such as a medical problem as a lower exercise tolerance on life was connected with lower quality of life in the areas of physical functioning, limiting roles—physical problems, social functioning but also with a lower result in physical dimension of quality of life, and general index of quality of life. Lower exercise tolerance may also be connected with a faster fatigue and greater tiredness. In the study by Peters et al. [16], 89% people of with Marfan syndrome reported a higher level of fatigue, and reports from Velvin et al. [22] show that lower satisfaction from life was connected with severe fatigue. Some studies state that ophthalmic problems worsen a patient’s state of mind and their satisfaction from life [31,32,33]. The authors of another group of studies [10,11,15,17,34] do not confirm such a relation in the case of patients with Marfan syndrome. Therefore, the result of our study is interesting; it confirms a lack of significant differences between objective data from the area of ophthalmic problems and quality of life, and the only significant relationship occurred in the evaluation of the subjective effect of these types of problems on life. The evaluation of a greater disorganizing effect of ophthalmic problems on life was connected with a lower quality in the area of vitality. The ophthalmic symptoms are connected with the discomfort related to the appearance and inability to do daily activities easily. It seems that the cognitive assessment of ophthalmic problems and giving them meaning as limiting may block the activity of people with Marfan syndrome, causing them to resign from participation in various activities, which might be connected with a lower evaluation of their quality of life in the area of vitality.

The evaluation of a greater disorganizing effect of the cardiological problems on life was connected with a lower quality of life in the area of limiting roles—physical problems. However, the objective clinical cardiological data reveal that people who do not take beta-blockers had a higher quality of life in the area of vitality. Another difference concerning the medical data from the cardiological area was observed in the scope of having a dilated aorta—people who did not have this symptom were characterized by a higher quality of life in the scope of state of mind.

People whose family members did not have an aorta dissection or who experienced no deaths have a higher quality of life in the area of physical functioning (differences at the level of statistical tendency) than people having such experiences. In the study by Peters et al. [16], it was indicated that aorta dissection in the past in a given person had a strong effect on the perception of the disease as negatively affecting life. Aorta dissection, experiencing joint pain, and depression were connected with the feeling of a lack of control over the disease and having a more pessimistic approach to treatment. During the patient’s education, it is worth focusing on these issues in order to precisely explain the mechanism of action of the process of aorta dissection and available possibilities of help, as well as in cases where beta-blockers are recommended. It seems that patients in cases of lack of information or insufficient understanding of the process of treatment may wrongly interpret their health status to their disadvantage, which may be related to a lowered quality of life. Analyzing the data from the results of our research on the correlation of various areas of quality of life and the subjective assessment of the patient’s impact of individual health problems on his life, it was noted that the subjective assessment of the impact of health status shows a large number of relationships with various areas of quality of life, which complements the information from a review by Nielsen et al. [35].

Moreover, as a result of our studies, similar to other studies [12,13,17,35], the following practical conclusions could be drawn. The improvement of the quality of life of patients with Marfan syndrome requires comprehensive support, assessment, and implementation of the support in the context of bio-behavioral factors, social support, and factors connected with the disease. If the patient can manage the bio-behavioral factors better, his/her quality of life can be improved. Bio-behavioral factors may be applied in the patient through consideration of factors connected with the disease. The results of people who have had a hernia and who were characterized by a higher evaluation of the quality of life in the area of vitality were interesting, but at the same time difficult to interpret. People in whom three or more dysmorphic facial features were observed had a higher quality of life in the area of vitality and mental health dimension.

In this situation, it is worth considering the following attempt to explain that the patients who have visible features of the disease receive more support from the environment. Additionally, when the diseases have such symptoms as hernias, which are the symptoms qualified for medical intervention, patients receive more attention from health care and more often have the feeling that actions towards the improvement of their health status have been undertaken.

## 5. Conclusions

1. The quality of life of people with MFS is lower in comparison with the quality of life of healthy people.
2. In organizing help for patients with MFS, attention should be paid to the differentiations in terms of gender, which was not found in the healthy group.
3. Patients’ beliefs about the impact of cardiac symptoms on their life is very important, and it should be the basis of medical evaluation, treatment, rehabilitation, and psychoeducational programs.
4. Psychological evaluation of patients for anxiety and depression and pain management and fatigue should be included, as research shows that these are important factors in the quality of life of patients.
5. It is worthwhile to search for the causes of fatigue and its relationship with reduced exercise tolerance, which will allow for the design of a patient care strategy.
6. The development of psycho-educational intervention programs may improve the quality of social support, and this in turn may affect the lowering bio-behavioral factors such as depression, which would constitute a good strategy for the improvement of the quality of life of the patients with Marfan syndrome.

## Figures and Tables

**Table 1:** Demographic data concerning both studied groups.

Variable	People with Marfan Syndrome (N = 35)		Healthy People (N = 33)	
	M	SD	M	SD
Age	32.68	11.97	33.03	11.95
	N	%	N	%
Sex:				
Women	21	60	21	63.64
Men	14	40	12	36.36
Professional activity:				
Professionally active	11	31.43	5	15.15
Professionally inactive	23	65.71	26	78.79
Education:				
Basic or vocational	3	5.57	1	3.03
Secondary	16	45.71	11	33.33
Higher	15	42.86	20	60.61
Residence:				
Village	10	28.57	2	6.06
Town > 30,000 inhabitants	6	17.14	5	15.15
City 30,000–100,000 inhabitants	8	22.86	7	21.21
City over 100,000 inhabitants	10	28.57	17	51.51
Relationship:				
People in a steady relationship	20	57.14	21	63.64
People not in a relationship	14	40	9	27.27
Financial situation				
Above the average	9	25.71	21	63.63
Average	19	54.26	10	30.30
Rather bad or bad	5	2.86	1	3.03
Membership of Marfan Poland Association				
Yes	18	60		
No	12	40		

**Table 2:** Clinical data concerning people with Marfan syndrome.

Variable	Number
Marfan syndrome:	
de novo	7
familial	15
Aorta condition:	
Dissected aorta	19
Non-dissected aorta	8
Result from the upper part of the norm	2
Dissection or rupture of aneurysm	3
The course of the disease:	
With a cardiac surgery	7
Without a cardiac surgery	21

**Table 3:** Medical data concerning Ghent criteria in people with Marfan syndrome.

Ghent Criteria	M
Total number of points in Ghent scale	7.63
	n
Thumb or wrist symptoms:	
Thumb symptom	22
Wrist symptom	19
Symptoms connected with chest:	
Pigeon chest	16
Pectus excavatum	8
Chest asymmetry	22
Symptoms connected with feet:	
Metatarsal deformation	7
Flat foot	14
Both criteria fulfilled	5
Pneumothroax	1
Reduced relation of the upper body part to the lower part, increased relation of the arms span to the height with the absence of scoliosis USLS < 85	28
Scoliosis, kyphosis	26
Limited strengthening of an elbow (<170°)	3
Dysmorphic facial features (≥3 z 5)	14
Stretch marks	16
Myopia > 3 Dioptres	17
Mitral valve prolapse	20

**Table 4:** The significance of the differences between a group with Marfan syndrome and a group of healthy people in the scope of the quality of life conditioned by the health status.

Quality of Life Conditioned by the Health Status (SF-36)	Marfan Syndrome		Healthy People		Significance of Differences		95% CI	
	M	SD	M	SD	t	*p*	LL	UL
PF	74.85	16.93	93.03	9.60	−5.37	0.000	−24.95	−11.41
RP	56.82	37.64	83.33	27.72	−3.26	0.002	−42.77	−10.26
BP	54.88	21.33	57.58	23.15	−0.49	0.625	−13.64	8.25
GH	29.85	19.54	53.18	17.85	−5.06	0.000	−32.54	−14.13
VT	53.03	14.57	60.91	18.81	−1.90	0.062	−16.15	0.40
SE	71.21	18.88	62.88	25.09	1.52	0.132	−2.59	19.25
RE	69.70	40.28	81.82	34.45	−1.31	0.194	−30.55	6.31
MH	64.97	14.66	69.21	14.19	−1.19	0.237	−11.34	2.85
PCS	49.15	8.41	58.76	6.49	−5.20	0.000	−13.30	−5.91
MCS	48.64	7.06	50.97	8.40	−1.22	0.226	−6.15	1.48
GS SF-36	97.79	14.06	109.73	13.86	−3.47	0.000	−18.81	−5.07

Note: PF—Physical Functioning, RP—Role-physical, BP—Bodily Pain, GH—General Health, VT—Vitality, SF—Social Functioning, RE—Role-emotional, MH—Mental Health, PCS—Physical Component Summary, MCS—Mental Component Summary, GS—General Scale.

**Table 5:** Comparison of mean results obtained in different areas of the quality of life in both studied groups with regards to gender.

Quality of Life Conditioned by the Health Status (SF-36)	Marfan Syndrome						Healthy People					
	K		M		Significance of Differences		K		M		Significance of Differences	
	M	SD	M	SD	t	*p*	M	SD	M	SD	t	*p*
PF	66.50	13.19	87.69	13.94	−4.41	0.000	91.43	9.24	95.83	9.96	−1.28	0.210
RP	57.50	34.51	55.77	43.49	0.13	0.900	80.95	27.28	87.50	29.19	−0.65	0.523
BP	55.56	23.08	53.85	19.16	0.22	0.826	54.50	20.76	62.96	26.94	−1.01	0.320
GH	24.25	17.34	38.46	20.25	−2.15	0.039	52.62	16.25	54.17	21.09	−0.24	0.815
VT	46.50	10.27	63.08	14.80	−3.81	0.001	59.76	18.20	59.76	18.20	−0.46	0.650
SE	69.38	20.06	65.00	43.90	−0.69	0.497	59.76	18.20	69.79	18.04	−1.21	0.237
RE	65.00	43.90	76.92	34.39	−0.83	0.415	77.78	37.02	88.89	29.59	−0.89	0.381
MH	60.20	15.65	72.31	9.45	−2.50	0.018	68.00	15.28	71.33	12.40	−0.64	0.525
PCS	46.45	6.99	53.31	8.97	−2.46	0.020	57.95	5.67	60.17	7.78	−0.94	0.354
MCS	45.85	6.29	52.92	6.13	−3.19	0.003	50.00	9.31	52.67	6.54	−0.87	0.389
GS SF-36	92.30	11.20	106.23	14.19	−3.14	0.004	107.95	14.40	112.83	12.87	−0.97	0.339

Note: PF—Physical Functioning, RP—Role-physical, BP—Bodily Pain, GH—General Health, VT—Vitality, SF—Social Functioning, RE—Role-emotional, MH—Mental Health, PCS—Physical Component Summary, MCS—Mental Component Summary, GS—General Scale.

**Table 6:** Correlations of a subjective assessment of the effects of health problems on life in the evaluation of people with Marfan syndrome and the quality of life.

SF-36	PF	RP	BP	GH	VT	SE	RE	MH	PCS	MCS	GS SF-36
Orthopedic problems	−0.72 **	−0.10	0.17	−0.58 *	−0.71 *	−0.54 *	−0.51	−0.52 *	−0.61 *	−0.76 **	−0.73 *
Ophthalmic problems	−0.34	0.05	0.31	−0.39	−0.57 *	−0.19	−0.20	−0.15	−0.21	−0.46	−0.36
Cardiological problems	−0.37	−0.61 *	0.07	−0.33	−0.11	−0.19	−0.49	−0.28	−0.42	−0.35	−0.41
Lower exercise tolerance	−0.52 *	−0.68 *	−0.23	−0.34	−0.39	−0.54 *	−0.34	−0.20	−0.56 *	−0.49	−0.56 *

Explanation: * *p* < 0.05; ** *p* < 0.01;. Note: PF—Physical Functioning, RP—Role-physical, BP—Bodily Pain, GH—General Health, VT—Vitality, SF—Social Functioning, RE—Role-emotional, MH—Mental Health, PCS—Physical Component Summary, MCS—Mental Component Summary, GS—General Scale.

**Table 7:** Correlation of the obtained Ghent points and a quality of life.

SF-36	PF	RP	BP	GH	VT	SE	RE	MH	PCS	MCS	GS SF-36
Number of Ghent points	−0.07	0.17	0.19	−0.35	−0.33	0.00	−0.21	−0.14	−0.20	−0.13	−0.18

Note: PF—Physical Functioning, RP—Role-physical, BP—Bodily Pain, GH—General Health, VT—Vitality, SF—Social Functioning, RE—Role-emotional, MH—Mental Health, PCS—Physical Component Summary, MCS—Mental Component Summary, GS—General Scale.

## Data Availability

The data are collected in a database prepared by the research team.
